# Plaque reduction neutralization antibody test does not accurately predict protection against dengue infection in Ratchaburi cohort, Thailand

**DOI:** 10.1186/1743-422X-11-48

**Published:** 2014-03-12

**Authors:** Chukiat Sirivichayakul, Arunee Sabchareon, Kriengsak Limkittikul, Sutee Yoksan

**Affiliations:** 1 Department of Tropical Pediatrics, Faculty of Tropical Medicine, Mahidol University, Bangkok, Thailand; 2 Center for Vaccine Development, Mahidol University, Nakhonpathom, Thailand

**Keywords:** Dengue, Plaque reduction neutralization test, Neutralizing antibody

## Abstract

**Background:**

The plaque reduction neutralization test (PRNT) is currently the best and most widely accepted approach to measuring virus-neutralizing and protective antibodies to dengue virus, and in assessing the immunogenicity of a dengue vaccine. However, the correlation between presence of dengue-neutralizing antibody and protection from infection is not absolute.

**Findings:**

In a cohort study in Ratchaburi Province, Thailand, 48 subjects with serologically confirmed symptomatic dengue infection were tested for pre-existing dengue neutralizing antibody using PRNT. Nine subjects had quite high pre-existing PRNT50 titers (titer >90) to subsequent infecting dengue serotypes, but still had symptomatic infections.

**Conclusion:**

This report provides evidence that PRNT may not be a good test for predicting protection against subsequent dengue infection.

## Findings

### Background

The plaque reduction neutralization test (PRNT) is a method for measuring antibodies that neutralize and prevent virions from infecting cultured cells. It is currently the most virus-specific serological test among the flaviviruses, and serotype-specific test among the dengue viruses [1]. PRNT has been widely used in assessing the protective neutralizing antibody response for Japanese-encephalitis vaccines [2-4].

For dengue, PRNT is the best and most widely accepted approach to measuring virus-neutralizing and protective antibodies [1], and assessing the immunogenicity of dengue vaccine [5-8]. However, the correlation between the presence of virus-neutralizing antibody and protection from infection is not absolute. This report aims to provide additional data on the correlation of pre-existing dengue-neutralizing antibody and protection from subsequent dengue infection.

## Methods

In the cohort study conducted among school children in Ratchaburi Province, Thailand [9], we prospectively collected serum samples annually from all subjects. Acute and convalescent serum samples were also collected from each febrile subject, irrespective of clinical diagnosis. Clinical diagnosis was performed by a pediatrician who was unaware of the dengue diagnostic test results. Clinical diagnoses of dengue fever (DF), dengue hemorrhagic fever (DHF), and DHF severity were made using the WHO criteria (1997) [10]. All blood samples were drawn into serum separator tubes, allowed to clot at room temperature for 1–2 hours, then stored at 4°C. Sera were separated into aliquots within 24 hours and stored at −70°C until laboratory testing. Dengue diagnostic testing was performed at the Center for Vaccine Development, Institute of Molecular Biosciences, Mahidol University, Salaya, Nakhonpathom, Thailand (CVD). Acute and convalescent sera were tested for dengue-virus-specific IgM/IgG by enzyme-linked immunosorbent assay (ELISA) using slightly modified method from that described previously [11]. The sensitivity of this test was 97% in paired sera [11]. An IgM anti-dengue level ≥ 1 unit in acute serum, or seroconversion of either IgM or IgG in paired sera, was considered indicative of acute dengue infection. Primary dengue infection was diagnosed when the IgM:IgG ratio was >1:1.8. Serum samples from acute dengue cases were tested for dengue-virus serotype by inoculation into *Toxorhynchites splendens* mosquitoes with immunofluorescence detection and serotyping [12].

We randomly selected 48 subjects with acute dengue infection in the year 2006. Pre-infection sera were retrieved from the previous annual serum samples and tested for pre-existing dengue- and Japanese encephalitis-neutralizing antibody using PRNT, as described by Russell *et al.*[13]. In the tests, conducted at the CVD, monkey kidney-derived LLC-MK2 cells were used for virus production and PRNT. The dengue viruses (D) used in the assay were D1 (16007), D2 (16681), D3 (16562), and D4 (1036). LLC-MK2 cells were seeded in 6-well plates at 1 × 10^5^ cells/well, and incubated for 6–8 days. Neutralizing sera were diluted to 1:5, followed by ten-fold serial dilutions using phosphate buffer solution (PBS) pH 7.5 with 30% fetal bovine serum, mixed with virus (for a final starting dilution of 1:10), and incubated. Following infection, cells were overlaid with 3.0% carboxymethyl cellulose with neutral red added. Plaques were visualized and counted after cultivation for 7 days. Data were interpreted using the Probit model with the SPSS program, and PRNT endpoint titers were expressed as the reciprocal of the last serum dilution. The PRNT titer was calculated based on a 50% reduction in plaque count (PRNT50).

## Results

Tables 1, 2, 3 show the pre-existing dengue PRNT50 titers in the sera of subjects in February 2006, date of subsequent dengue illness, clinical diagnosis, and the serotype isolated. Among 48 subjects with serologically confirmed dengue infection, dengue viruses could be identified in 31 (64.6%) subjects, comprising 16 D1; 1 D2; 3 D3; and 11 D4. Only 5 (10.4%) subjects had primary infections.

**Table 1:** Pre-existing PRNT50 titer and subsequent dengue infection in subjects with low titer (<90) to subsequent infecting serotype

**Subject code**	**PRNT50 titer (Feb 2006)**					**Date of illness**	**Clinical diagnosis**	**ELISA test result**^**a**^	**Serotype isolated**
	**D1**	**D2**	**D3**	**D4**	**JE**				
03-146	<10	<10	<10	<10	155	11/10/2006	DF	Secondary	D1
05-119	<10	<10	<10	<10	235	9/6/2006	Pharyngitis	Secondary	D1
05-181	<10	<10	<10	<10	<10	21/8/2006	DF	Primary	D1
05-310	10	<10	167	<10	250	21/4/2006	DF	Secondary	D1
07-479	<10	<10	<10	<10	29	23/7/2006	DF	Secondary	D1
07-383	13	<10	<10	<10	<10	10/9/2006	DF	Primary	D1
04-276	13	<10	<10	<10	<10	13/3/2006	DF	Primary	D1
05-357	40	29	27	<10	303	10/10/2006	Bronchitis	Secondary	D1
05-002	50	<10	<10	<10	396	25/11/2006	DF	Secondary	D1
03-097	75	1134	24	20	1307	7/10/2006	DF	Secondary	D1
05-339	<10	<10	<10	<10	503	28/8/2006	Pharyngitis	Secondary	D2
04-322	17	<10	<10	<10	165	9/11/2006	DF	Secondary	D3
04-325	<10	<10	<10	<10	685	23/10/2006	DHF gr2	Secondary	D3
02-189	<10	10	12	<10	<10	6/7/2006	DF	Primary	D3
01-227	49	<10	<10	<10	590	19/2/2006	Pharyngitis	Secondary	D4
01-254	12450	3348	32	<10	78	28/2/2006	AGE	Secondary	D4
01-384	<10	<10	<10	<10	92	19/12/2006	Pharyngitis	Secondary	D4
06-164	210	540	12040	21	76	30/8/2006	DF	Secondary	D4
01-124	228	135	516	39	28	31/3/2006	DF	Secondary	D4
05-257	3141	194	272	41	726	15/10/2006	Common cold	Secondary	D4
01-224	195	2901	220	50	802	9/3/2006	DHF gr1	Secondary	D4
05-378	6238	2204	676	75	43	28/7/2006	DF	Secondary	D4

^a^ELISA result showed either primary or secondary infection.

AGE: acute gastroenteritis; D: dengue virus; DF: dengue fever; DHF: dengue hemorrhagic fever; gr: grade; JE: Japanese encephalitis virus; PRNT50: 50% plaque reduction neutralization.

**Table 2:** Pre-existing PRNT50 titer and subsequent dengue infection in subjects with high titer (>90) to subsequent infecting serotype

**Subject code**	**PRNT50 titer (Feb 2006)**					**Date of illness**	**Clinical diagnosis**	**ELISA test result**^**a**^	**Serotype isolated**
	**D1**	**D2**	**D3**	**D4**	**JE**				
06-043	121	224	83	<10	11	14/8/2006	DF	Secondary	D1
05-021	133	<10	<10	<10	72	26/4/2006	DF	Secondary	D1
05-074	173	1136	73	31	503	9/5/2006	DF	Secondary	D1
06-082	317	<10	18	<10	760	14/6/2006	Viral infection	Secondary	D1
01-286	581	<10	<10	<10	<10	19/4/2006	DF	Secondary	D1
01-141	1848	821	4521	328	738	6/11/2006	Pharyngitis	Secondary	D1
07-119	5291	84	332	98	207	28/7/2006	DF	Secondary	D4
05-244	4916	1305	1006	145	393	21/9/2006	DF	Secondary	D4
04-378	11682	853	417	261	5615	5/7/2006	DF	Secondary	D4

^a^ELISA result showed either primary or secondary infection.

D: dengue virus; DF: dengue fever; JE: Japanese encephalitis virus; PRNT50: 50% plaque reduction neutralization.

**Table 3:** Pre-existing PRNT50 titer and subsequent dengue infection among subjects whose subsequent infecting serotypes could not be identified

**Subject code**	**PRNT50 titer (Feb 2006)**					**Date of illness**	**Clinical diagnosis**	**ELISA test result**^**a**^
	**D1**	**D2**	**D3**	**D4**	**JE**			
01-177	537	218	619	74	14	1/6/2006	Pharyngitis	Secondary
01-437	473	297	1283	<10	75	9/9/2006	Influenza	Secondary
01-456	14105	2851	7872	235	594	27/6/2006	DF	Secondary
01-559	393	1606	249	65	4987	14/8/2006	viral infection	Secondary
02-246	<10	<10	16	<10	2201	3/5/2006	DF	Secondary
02-434	<10	<10	<10	<10	1326	31/3/2006	Pharyngitis	Secondary
02-453	172	2053	126	53	14587	10/3/2006	DHF gr3	Secondary
03-021	867	<10	1026	<10	<10	29/11/2006	DHF gr1	Secondary
05-072	351	36	<10	174	140	1/12/2006	Viral infection	Secondary
05-0112	<10	<10	<10	<10	<10	2/12/2006	URI	Primary
05-209	310	48	753	14	76	29/7/2006	DHF gr1	Secondary
05-239	3257	8199	111	40	919	24/4/2006	DHF gr1	Secondary
05-358	249	247	3934	34	267	10/6/2006	Viral infection	Secondary
06-070	236	2687	10649	<10	94	16/6/2006	Pharyngitis	Secondary
06-124	472	5074	3943	29	164	27/8/2006	DHF gr1	Secondary
06-192	10	<10	<10	<10	42	26/8/2006	Pharyngitis	Secondary
07-310	137	43	23	198	21	7/8/2006	Influenza	Secondary

^a^The ELISA result showed either primary or secondary infection.

D: dengue virus; DF: dengue fever; DHF: dengue hemorrhagic fever; gr: grade; JE: Japanese encephalitis virus; PRNT50: 50% plaque reduction neutralization.

Of 31 subjects whose infecting dengue serotypes were identified, 14 (45.2%) had pre-existing PRNT50 titers to the infecting serotype < 20. Eight subjects (25.8%) had pre-existing PRNT50 titers to the infecting serotype of between 21 and 75 (Table 1).

Interestingly, nine (29.0%) subjects had pre-existing PRNT50 titers of > 90 to the subsequent infecting serotypes. Six subjects with D1 infections had pre-existing PRNT50 titers to D1, which ranged from 121 to 1848; geometric mean value 313; median value 245. Two subjects (subjects 01–286 and 05–021) had PRNT50 profiles suggesting previous primary D1 infection, but had secondary symptomatic infections with the same serotype. Three subjects with D4 infections had pre-existing PRNT50 titers to D4, ranging between 98 to 261, geometric mean value 154 (Table 2).

It is worth noting that many subjects (e.g. subjects 01–456 and 04–378) had pre-existing PRNT50 profiles suggesting secondary dengue infection, but still had symptomatic infections, which were probably tertiary infections (Tables 3 and 2, respectively).

## Discussion

Dengue viruses comprise 4 serotypes. Infection with one dengue serotype elicits lifelong homotypic immunity, but only short-lived immunity for heterotypic serotypes [14]. Dengue neutralizing antibody has been believed to represent protection against dengue, and the PRNT test has been widely used to measure this neutralizing antibody. Numerous vaccine immunogenicity assessment laboratories consider a seropositive threshold to be 10 [1] and since four of the subjects in this report had PRNT50 titers of 10–13, we arbitrarily divided the subjects into 3 groups, i.e. titer <20, 20–90, and >90. We found that 17 (54.8%) and 9 (29.0%) of 31 subjects had pre-existing PRNT50 titers >20 and >90, respectively, to the subsequent infecting dengue serotype. These data provide partial insight into the correlation between PRNT50 titer and disease protection. This is very important, because PRNT titer is considered an important marker of protection in the development of dengue vaccines. These data are perhaps the most relevant available data, as more valid data on the correlation between pre-existing PRNT50 titer and disease protection in humans requires human challenge with dengue virus, which may not be possible due to ethical issues. This report raises some inconsistencies with our previous understandings. First, the finding in 2 subjects (subjects 01–286 and 05–021 [Table 2]) suggests that previous D1 infection may not induce protection to subsequent symptomatic homotypic dengue infection. Second, a quite high pre-existing PRNT50 titer (>90) may not be able to protect against subsequent symptomatic infection from the respective dengue serotype.

In a cohort study in Thailand, Endy *et al*. [15] also found that pre-existing neutralizing antibody directed against infecting dengue serotype (titer >10) was detected in 36%, 67%, and 46% of D3, D2, and D1 infections, respectively. Moreover, only a pre-existing PRNT50 > 100 against the reference D3 strain was associated with milder severity of disease, but not in D2 and D1. This is further confirmed by the finding in a phase-2b dengue-vaccine trial among Thai children that the tetravalent live-attenuated dengue vaccine had a low level of efficacy against D2, despite its high immunogenicity [16].

There are some possible explanations for the lack of a definite correlation between PRNT50 titer and protection from subsequent dengue infection. One possible explanation is that in our PRNT, we used LLC-MK2 cells, which are not FcγR-expressing cells. In the absence of FcγR, dengue virus-antibody complexes are not able to infect the cells, while these complexes are taken up more efficiently by FcγR-expressing cells, and are still infectious [17]. This is supported by the study of Moi *et al.*[18], who found that 11 of 18 serum samples from patients with acute secondary dengue infection demonstrated neutralizing activity to the infecting serotype, determined using FcγR-negative BHK cells, but not when determined using FcγR-expressing cells. Another explanation is that the protective PRNT50 titer for dengue may be much higher than the titer of 10, defined for Japanese encephalitis virus, and the protective level of dengue neutralizing antibody should be more accurately defined. This study revealed that subjects with pre-existing PRNT50 titer of up to 1848 against D1, and 261 against D4, still had symptomatic infections due to the respective serotypes, suggesting the protective level should be higher and may differ for different serotypes. Nevertheless, defining the protective-level cut-off point is difficult and challenging. A very large cohort study and long-term follow-up are needed, unless a challenge test in subjects with pre-defined PRNT levels could be conducted. Moreover, as PRNT titers vary significantly depending on testing conditions, such as virus strains, virus passage and cell type [19,20], optimal testing conditions should be defined.

Finally, the pre-infection PRNT50 titers are against reference dengue-virus strains. As molecular evolution among dengue viruses has been continuous [21], it may cause antigenic mismatches between the reference dengue virus strains used in the PRNT and infecting viruses, and therefore, mismatch between the pre-existing antibody and the antigen of the infecting homologous serotype. Further studies are needed to clarify these possibilities.

It is also noted that dengue-naïve but Japanese encephalitis (JE)-immuned subjects shown by PRNT (e.g. subjects 01–384, 04–325, 07–479) showed secondary antibody response to subsequent dengue infection. One subject (subject 04–325) had DHF grade2. These pieces of evidence suggest cross-reactive antibody responses between dengue and JE.

## Competing interests

The authors declare that they have no competing interests.

## Authors’ contributions

CS, AS and KL designed the study, collected data and specimens, wrote and reviewed the manuscript. SY performed laboratory tests, wrote and reviewed the manuscript. All authors read and approved the final manuscript.
